# The Place of the Hospitals

**Published:** 1918-12-07

**Authors:** 


### THE PLACE OF THE HOSPITALS.

### Address of the Archbishop of Canterbury to Hospital Workers.

His Grace said he wished to take as text or motto some words which seemed to him singularly appropriate to gatherings such as this, alike in its character and its purpose: "Christ, from whom the whole body fitly joined together and compacted by that which every joint supplieth . . . maketh increase of the body unto the building up of itself in love." A vigorous, most capable man, perhaps one of the most notable and vivid personalities in a sordid world, was writing, towards the end of his life, from a prison in Rome, telling a new message to the peoples, or some of them, in the great cities of the world. It was a message about a new beginning, a fresh start, for human life, a new proclamation of an universal sort. For the letter, the Epistle to the Ephesians, was, as its original showed, a circular-letter: it was written for others besides the men and women of Ephesus. The message was given out at a great crisis—the greatest, perhaps, till now, in the world's story—the crisis hour when the dominant world-forces, the culture of Greece, the law and orderliness of Rome, were on the wane, and the torch of a new light was flashing across the world, from Jerusalem to Antioch, from Antioch to Venice, from Venice to Imperial Rome. The note of the new message was the note of unity in service. The message told in that crisis of human history he told now, certainly the greatest crisis time since then, and his Grace and the people generally were those to realise it at this crisis time and use its opportunities, as they were trying to do.

### Christ's Message.

The gist of the message was, that God meant those people who were in earnest to be welded into a society, a body, which would be largely responsible for the welfare of the world; and Christ, in His message given forth by St. Paul, showed us how. That message said that we were part of a society of men—a living, pulsing thing; we were fashioning it, or could fashion it, into a stronger force. That society was an organism comprising all varieties of material going to make up the body corporate. It had one link or bond, one cement which united it, one personal Example, its living Parent. That sense of His Person would be more readily and constantly felt and expressed by some persons than by others; but if this earnestness, self-sacrifice, and devotion were traced to its very source—remembering the environment in which workers were placed to-day and the beginnings of that environment—its most vital centre would be found to be in the life and human environment of Christ, from whom the whole world, fitly joined together and compacted by that which every joint supplieth, maketh increase of the body unto the building up of itself in love. That was the message of the scarred, travel-worn, eager old man nearly 1,900 years ago. It came flashing into those busy, crowded, slave-cursed cities around the Mediterranean, the cities of the Roman Empire.

### Not Apart, but Part.

That seemed a long way from us, gathered in London under conditions so different, gathered, apparently, apart in our human work and activity. But it was not apart, it was close. The principle which that far-seeing message laid down still held good here and now. There was a corporate loyalty now to that example which St. Paul gave, a fellowship, a devotion to service on his lines. Then there was the coherence on that basis, a coherence of even those men and women who did not regard themselves as being specially religious, either in origin or character. They were not apart, but part. That was the root of the counsel, however it might be regarded now. That society comprised all sorts of people; it might, in some lands, have a national character. But the real cement of the two was a loyalty to those principles of devotion and service, and the knowledge which was there.

### The Gospel of Repair and Recuperation.

He remembered being struck, years ago, by reading a lecture which had been delivered in the College of Surgeons by Sir James Paget, when he was professor, sixty or seventy years ago. The lecture was published later, somewhat changed in form. Its theme was the place of an active, recuperative, repairing agency which exists in every human body, the power of that agency to make good all sorts of injuries, even those which were rare and not to be expected, all because such a force was always there. It had often seemed to him that the picture—of which his Grace was not capable of estimating the value in its technical sense—drawn by a master hand, of something resident in the human body, corresponded very much to the place of our hospitals in the body of the community as a whole, what Paget called the gospel of repair and recuperation, something which was of use in our necessities and in our troubles. This thought, "always there," possessed a special significance just now. We had passed through a war far and away unparalleled, in the scale of its horrors, in the story of the human race. He supposed it was true to say that never was there, at one time, so vast a number of maimed and hurt people alive as to-day; and certainly there had never been so vast an arrangement for their relief. That was an object lesson which was patent to us everywhere. Hundreds of thousands of men, tens of thousands of workers, would live the rest of their lives with the thought upon them of what these years of suffering and of the treatment of suffering had meant for them. It was a new revelation to the womanhood of our land, from quarters quite unexpected; it was a new experience in the life of our citizens which they never thought to go through. And with that experience and

**The Archbishop of Canterbury's Address**—(*cont.*)

that knowledge we were going to make a new start now in the national and international life with those memories fresh upon us.

WORKERS LOYAL TO CHRIST'S PRINCIPLES.

With those memories and thoughts upon us, as we made use of them for good, more active, more coherent, more progressive, more hopeful and expectant in our common life, compacted by that which every joint supplieth, especially did that apply to such work as that which united those he was addressing that day. He dare not say for a moment that there was only one way in which that could be recognised as an united, compact action, but he believed it to be true that all wise effort of this sort could be traced back to one great Source. At all events, it was very markedly true in one way, even among those who did not recognise or acknowledge the source, the force, the secret of their energy; it did come from Christ, "making increase of the body in love." Speaking generally, the workers of the land were loyal to those principles, the principles which Christ taught in His life and in His death, the principle of service to the weak, service to the needy—whatever that might mean. The old Apostle St. John, years after Christ left this earth, looking back on the past, said "whoso hath this world's goods"—education, knowledge, good intentions—"and seeth his brother in need" of any of the bodily, mental, or spiritual requirements—"and shutteth up his compassion, how dwelleth the love of God in him?" He was basing that, as an old man, upon the recollection of how Christ said "Inasmuch as ye did it, inasmuch as ye did it not, unto one of the least of My brethren, ye did it, ye did it not, unto Me."

FREELY YE HAVE RECEIVED, FREELY GIVE.

With those thoughts we realised how that recuperative force which, in our hospitals, lay at the centre, remained as effective, as strong, as potent for body, mind, and soul, as we could ever make it. For that purpose we were compacted into one body, so that every set of people bore their part, "making increase of the body," making the whole thing stronger, by the "building up of itself in love." That love would tell in outward acts, and they resolved that they would make as efficient as ever they could that great recuperative force, the gospel of re-education and amelioration, to see that it was exemplified in our outward life in the hospital system. So could we go forth to-day with those memories upon us to work freely, having always in mind the words "Freely ye have received, freely give."

